# Emergency CT KUB: Incidental Findings and Their Clinical Significance

**DOI:** 10.2147/OAEM.S620255

**Published:** 2026-09-09

**Authors:** Mostafa Dayraki, Shahzad Anjum, Lolwa AlKhulaifi, Lara Miranda, Duha Adil Musa Mohmmad, Atika Jabeen, Zain Ali Bhutta, Yavuz Yigit

**Affiliations:** 1 Department of Emergency Medicine, Hamad Medical Corporation, Doha, Qatar; 2 College of Medicine, Weill Cornell Medicine-Qatar, Doha, Qatar; 3 College of Medicine, QU Health, Doha, Qatar; 4 Blizard Institute, Queen Mary University, London, UK

**Keywords:** CT KUB, incidental findings, emergency department, obstructive uropathy, risk stratification

## Abstract

**Background:**

Incidental findings (IFs) on computed tomography of the kidneys, ureters, and bladder (CT KUB) are increasingly common in emergency departments (EDs) due to the widespread use of advanced imaging. These findings, often unrelated to the primary diagnostic indication, present clinical and operational challenges, particularly in high-volume, demographically diverse settings.

**Objective:**

To evaluate the prevalence, classification, and clinical significance of incidental findings on CT KUB scans performed in the ED for suspected obstructive uropathy, and to identify factors associated with clinically significant IFs and evaluate their clinical outcomes.

**Methods:**

A retrospective single-center observational study was conducted at a national tertiary-care hospital in Qatar, including all adult patients who underwent non-contrast CT KUB for suspected renal colic between January 1 and November 30, 2024. Demographic, clinical, and radiological data were extracted. IFs were categorized as major, moderate, or minor based on clinical relevance. Outcomes included prevalence and distribution of IFs, subsequent management actions, and factors associated with major IFs.

**Results:**

Of 2786 CT KUB scans analyzed, 1531 (54.9%) revealed at least one incidental finding. Most incidental findings were classified as minor (63.2%), whereas 6.1% were major and 30.8% were moderate according to the highest-severity incidental finding per scan. Major IFs (e.g, suspected malignancy, aortic aneurysm) accounted for most urgent interventions and admissions. Multivariable analysis identified that older age, nationality, fever, desaturation, and abdominal tenderness were independently associated with major IFs (all p<0.05). Management of the primary condition was delayed due to IFs in only 0.6% of cases. The most frequent IFs were vascular calcifications, bone lesions, and hernias.

**Conclusion:**

Incidental findings on CT KUB are common in the ED, with a significant proportion requiring further intervention or specialist referral. The high prevalence in a younger, multi-ethnic population underscores the need for context-specific protocols, structured reporting, and robust follow-up systems.

## Introduction

Incidental findings (IFs) and unexpected abnormalities during diagnostic imaging have become increasingly common with the widespread use of advanced imaging technologies, particularly computed tomography (CT).^1^,^2^ These findings can range from benign anatomical variations to clinically significant lesions, including malignancies or vascular abnormalities.^3^,^4^ Computed tomography (CT) in the Emergency Department (ED) is often performed under urgent clinical conditions, where incidental findings pose a unique challenge by potentially diverting attention from the primary diagnostic target while introducing new concerns regarding follow-up, risk communication, and healthcare utilization.^5^ Although many findings are benign and require no intervention, a subset may demand further investigations, speciality consultations, or management, creating a significant burden on patient management and healthcare resources, particularly in the emergency setting.^6–9^

Non-contrast CT of the kidneys, ureters, and bladder (CT KUB) is the first-line imaging modality in evaluating suspected obstructive uropathy, including nephrolithiasis.^10^ CT KUB is highly sensitive and specific for detecting urinary tract stones and other obstructive causes,^11^ and is routinely performed in the ED for patients presenting with acute flank pain, hematuria, or urinary symptoms.^12^ Considering the increasing utilization and field of view for CT KUB, it often leads to the detection of incidental findings unrelated to the primary indication such as hepatic lesions, adrenal nodules, and bowel abnormalities.^3^,^13^,^14^

Systematic reviews and meta-analyses have reported that 30–50% of CT scans yield one or more incidental findings, depending on the anatomical region and patient population.^2^,^15^,^16^ Common incidental findings include simple renal cysts, uterine fibroids, liver lesions, vascular calcifications, pulmonary nodules, and occasionally masses suspicious for malignancy.^12^,^17–19^ The clinical dilemma lies in balancing the potential benefit of early detection of serious conditions against the risks of overdiagnosis, patient anxiety, unnecessary investigations, increased resource utilization, and treatment delays.^20^,^21^ The American College of Radiology (ACR) and other professional bodies have developed organ-specific classifications and management guidelines to assist clinicians in categorizing and managing incidental findings based on size, morphology, and patient risk factors.^3^,^15^,^22^ Nevertheless, considerable variability remains in how incidental findings are classified and reported across CT studies, limiting comparisons between studies and interpretation of their clinical significance.^23^,^24^ To our knowledge, few studies have applied such a hierarchical classification to emergency CT KUB examinations while simultaneously evaluating factors associated with incidental findings and their clinical outcomes. Furthermore, adherence to these guidelines in the emergency setting is inconsistent, and their applicability to diverse populations and healthcare systems remains limited.^25–27^

Despite growing global awareness of incidental findings, data from the Middle East, including Qatar is limited. Qatar’s demographically diverse population, along with a high volume of ED imaging, provides a unique setting to evaluate the prevalence and outcomes of incidental findings on CT KUB.^28^ A more comprehensive understanding of their patterns and their clinical implications has a potential to inform clinical practice, optimize resource utilization and guide future policies for the management of incidental imaging findings.

This study aims to evaluate the prevalence, classification and clinical significance of incidental findings on CT KUB scans performed in the Emergency Department. The study’s objectives are to describe the prevalence of incidental findings, classify them by anatomical site and clinical relevance, assess the proportion of follow up interventions and care pathways and identify demographic or clinical factors associated with clinically significant incidental findings.

## Methodology

### Study Design and Setting

We conducted a retrospective observational study in the Emergency Department of a national tertiary-care hospital in Qatar. The department is one of the largest and busiest in the region, managing approximately 400,000 patient visits annually.^29^ All non-contrast CT KUB examinations performed between 1 January 2024 and 30 November 2024 were eligible for inclusion. The study was approved by the local Institutional Review Board (IRB) (Hamad Medical Corporation, Medical Research Center, Protocol no: MRC-01-24-671). The requirement for written informed consent was waived due to the retrospective nature of the study and the use of de-identified clinical data.

### Study Population

**Figure 1:**
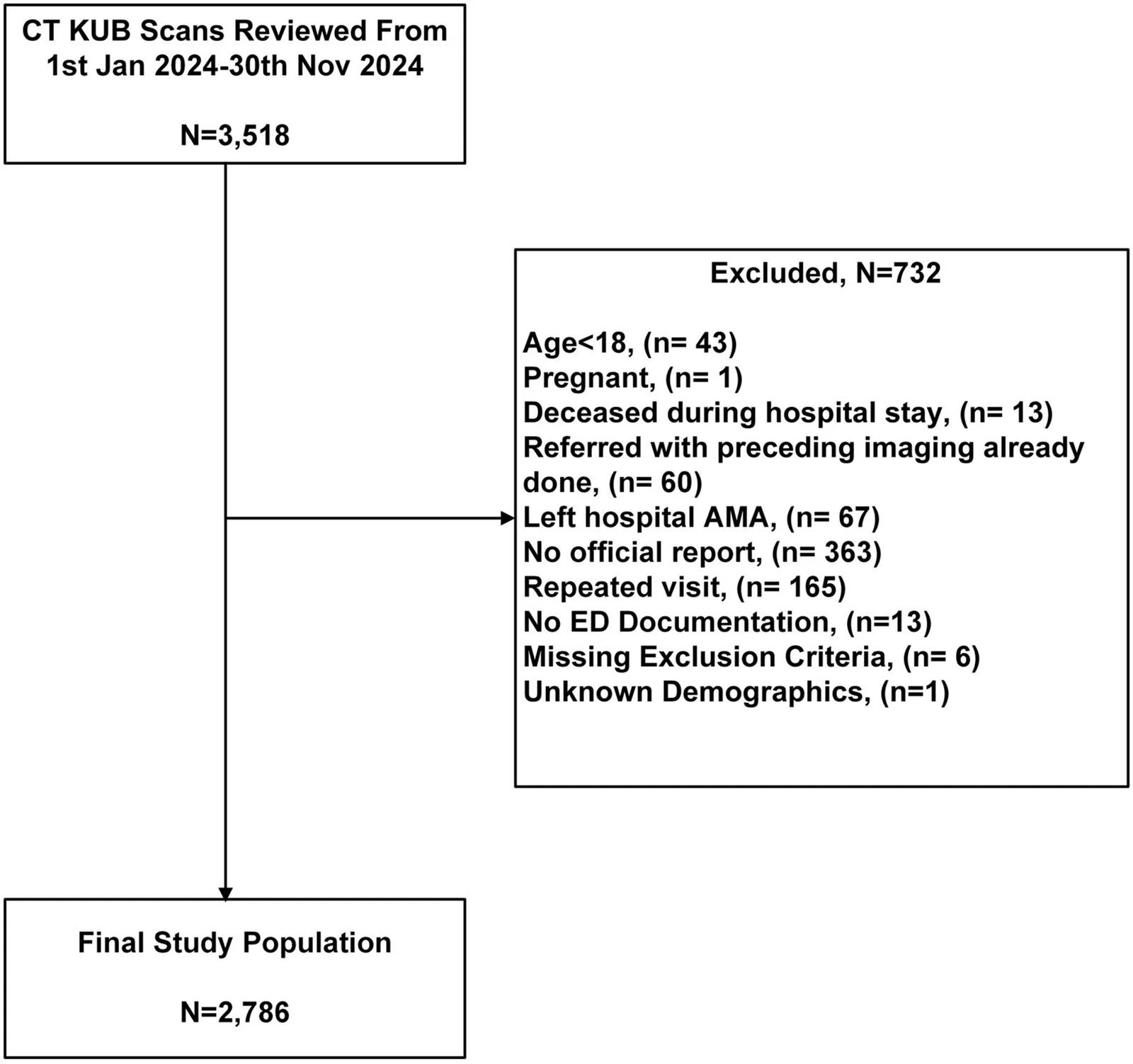
Flowchart of Patient Inclusion and Exclusion.

Inclusion criteria were: adult patients (≥18 years) who underwent non-contrast CT KUB in the emergency department for suspected renal colic during the study period. The following exclusion criteria were applied: 1) patients under the age of 18 years, 2) pregnancy, 3) death during hospital stay, 4) referred from other facilities with prior imaging, 5) patients left against medical advice, 6) cases lacking formal radiology reports by board-certified radiologists at the study center 7) for patients with repeated emergency department visits during the study period, only the first CT KUB examination was included in the analysis to ensure patient-level observations and 8) patients with no documented ED visit records. A flowchart summarizing patient inclusion and exclusion is presented in Figure 1.

### Data Collection and Definitions

Demographic and clinical data (age, sex, nationality, comorbidities, presenting symptoms) were extracted from the electronic medical record. Radiology reports were reviewed to identify the presence of urolithiasis and any incidental findings, defined as any abnormality unrelated to the suspected urinary tract stones. Findings not directly related to renal colic or obstructive urolithiasis (eg, appendicitis or other non-urological abdominal pathology) were classified as incidental findings.

The findings were categorized according to their clinical significance and potential impact on patient management, informed primarily by published American College of Radiology (ACR) recommendations,^3^,^22^ relevant literature, and investigator consensus. Major findings were defined as urgent abnormalities requiring prompt diagnostic evaluation, specialist consultation, or treatment during the index admission or shortly after discharge (eg, suspected malignancy or aortic aneurysm). Moderate findings were defined as non-emergent abnormalities requiring patient notification and further outpatient evaluation, surveillance, or management according to the clinical context (eg, complex renal cysts). Minor findings were defined as benign or low-risk abnormalities not requiring additional investigation or follow-up (eg, simple renal cysts or uterine fibroids). Incidental findings were classified on a case-by-case basis by three investigators using these predefined criteria. When uncertainty arose regarding the appropriate classification, the case was reviewed with a senior investigator until consensus was reached. For patient-level analyses, when more than one incidental finding was identified in the same patient, the patient was assigned to the category corresponding to the highest-severity incidental finding. Management actions were recorded as patient-level binary indicators and were not mutually exclusive; patients could receive more than one management action.

### Outcomes

The primary outcome was the prevalence of incidental findings. Secondary outcomes included the distribution of incidental finding categories (major, moderate, minor); subsequent clinical management (additional imaging, specialist referral, treatment initiation, admission, outpatient follow-up); and delay in the management of the primary diagnosis attributable to incidental findings observed. Delay was defined as postponement of management for the primary renal colic diagnosis due to additional diagnostic evaluation or treatment prompted by an incidental finding during the same emergency department visit.

### Statistical Analysis

Categorical variables were reported as frequencies and percentages. The distribution of continuous variables was assessed visually using histograms with superimposed normal density curves and summarized as means with standard deviations or medians with interquartile ranges (IQR), as appropriate. Multivariable logistic regression was used to evaluate factors independently associated with major incidental findings. The primary regression outcome was the presence of a major incidental finding, defined as a binary patient-level variable (≥1 major incidental finding vs none). Adjusted odds ratios (aORs) with 95% confidence intervals (CIs) were estimated.

Candidate covariates were selected based on prior literature and clinical plausibility and assessed using univariable logistic regression. Variables with p<0.25 were considered eligible for inclusion in the multivariable model, followed by backward elimination. Model discrimination and fit were evaluated using the area under the receiver operating characteristic curve (AUC), Akaike information criterion (AIC), and Bayesian information criterion (BIC). The final model retained statistically significant variables and clinically relevant variables whose inclusion contributed to overall model fit. Age was modeled continuously, and all other covariates were binary. Nationality was collapsed into Qatari and Middle Eastern Arab versus all other nationalities to improve model parsimony and reflect the demographic context of the study population. The final model included seven parameters and 93 outcome events, yielding approximately 13.3 events per parameter. A sensitivity analysis excluding nationality was performed to assess the robustness of associations.

Model assumptions and adequacy were assessed using the Box–Tidwell approach for linearity in the logit, variance inflation factors for multicollinearity, the link test for model specification, and the Hosmer–Lemeshow goodness-of-fit test. Internal validation using 1000 bootstrap resamples was performed to assess model optimism and calibration. Time to follow-up was evaluated using Kaplan–Meier analysis and the Log rank test. Missing data were minimal (<1% across variables) and handled using complete-case analysis. All analyses were performed using Stata/IC version 16.1 (StataCorp, College Station, TX, USA).

## Results

A total of 2786 CT KUB scans were analyzed (Table 1). The median age was 41 years (IQR 32–54), and 68.5% were male. The largest nationality group was Qatari (39.1%), followed by South Asians (23.3%) and Africans (16.3%).

**Table 1:** Baseline Characteristics of Study Population (N =2786)

Variable (N=2786)	Frequency (Percentage) n (%)
Age (Median, IQR)	41 (32.0, 54.0)
Male sex	1909 (68.5%)
Nationality	
Qatari	1089 (39.1%)
Middle Eastern Arabs	451 (16.2%)
South Asians	650 (23.3%)
Africans	455 (16.3%)
Filipino	80 (2.9%)
European	25 (0.9%)
Other Nationalities	36 (1.3%)
Hypertension	546 (19.6%)
Diabetes Mellitus	522 (18.7%)
Chronic Kidney Disease	130 (4.7%)
Cardiovascular Disease	186 (6.7%)
Flank Pain	2247 (80.7%)
Urinary Symptoms	1390 (49.9%)
RBC in Urinalysis	1201 (43.1%)
CVA Tenderness	445 (16.0%)
Triage Categories	
Triage Category 2	20 (0.7%)
Triage Category 3	1909 (68.5%)
Triage Category 4	798 (28.6%)
Triage Category 5	57 (2.0%)
Missing Triage Category	2 (0.1%)

**Abbreviations**: IQR, Interquartile range; RBC, Red Blood Cell Count; CVA, Costovertebral angle.

**Table 2:** Incidental Findings Categories and Distribution, Follow-Up Outcomes for Incidental Finding, and Management of Primary Finding Affected Due to Incidental Finding

Variables	Frequency	Percentage
**Incidental Findings**	1531/2786	54.9%
Major	93/1531	6.1%
Moderate	471/1531	30.8%
Minor	967/1531	63.2%
**Follow-up Outcomes for IF**		
Additional Imaging	48	3.1%
Treatment Initiated	103	6.7%
Any Referral	117	7.6%
Admission	56	3.7%
Scheduled Workup	31	2.0%
Routine Follow up	73	4.8%
Urgent Follow up	171	11.2%
Investigation (Other than Imaging)	38	2.5%
**Primary Finding Management**		
Primary condition management delayed due to IF	17	0.6%
Admission under another specialty for IF	412	14.8%

**Notes**: Severity categories of incidental findings are mutually exclusive and represent hierarchical classification by highest severity. In contrast, follow-up outcomes and management variables are non–mutually exclusive, as a single patient may have undergone multiple management actions.

**Abbreviation**: IF, Incidental Finding.

**Figure 2:**
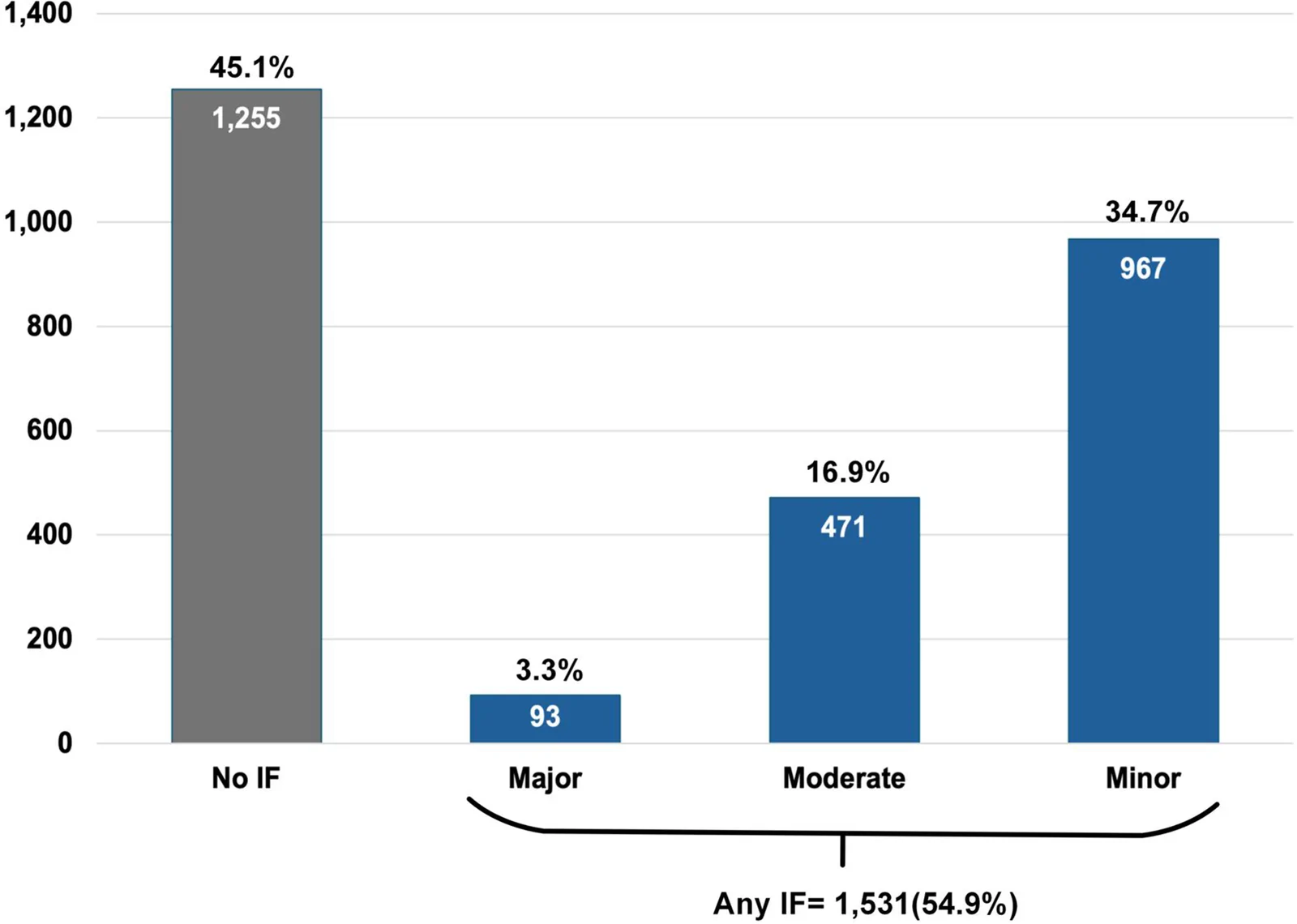
Distribution of Incidental Finding Severity Categories.

**Notes**: The bar chart shows the percentage distribution of patients with no incidental findings (IFs) and any IF, with incidental findings further classified according to the highest-order severity category (Major > Moderate > Minor). The y-axis represents the number of patients.

**Figure 3:**
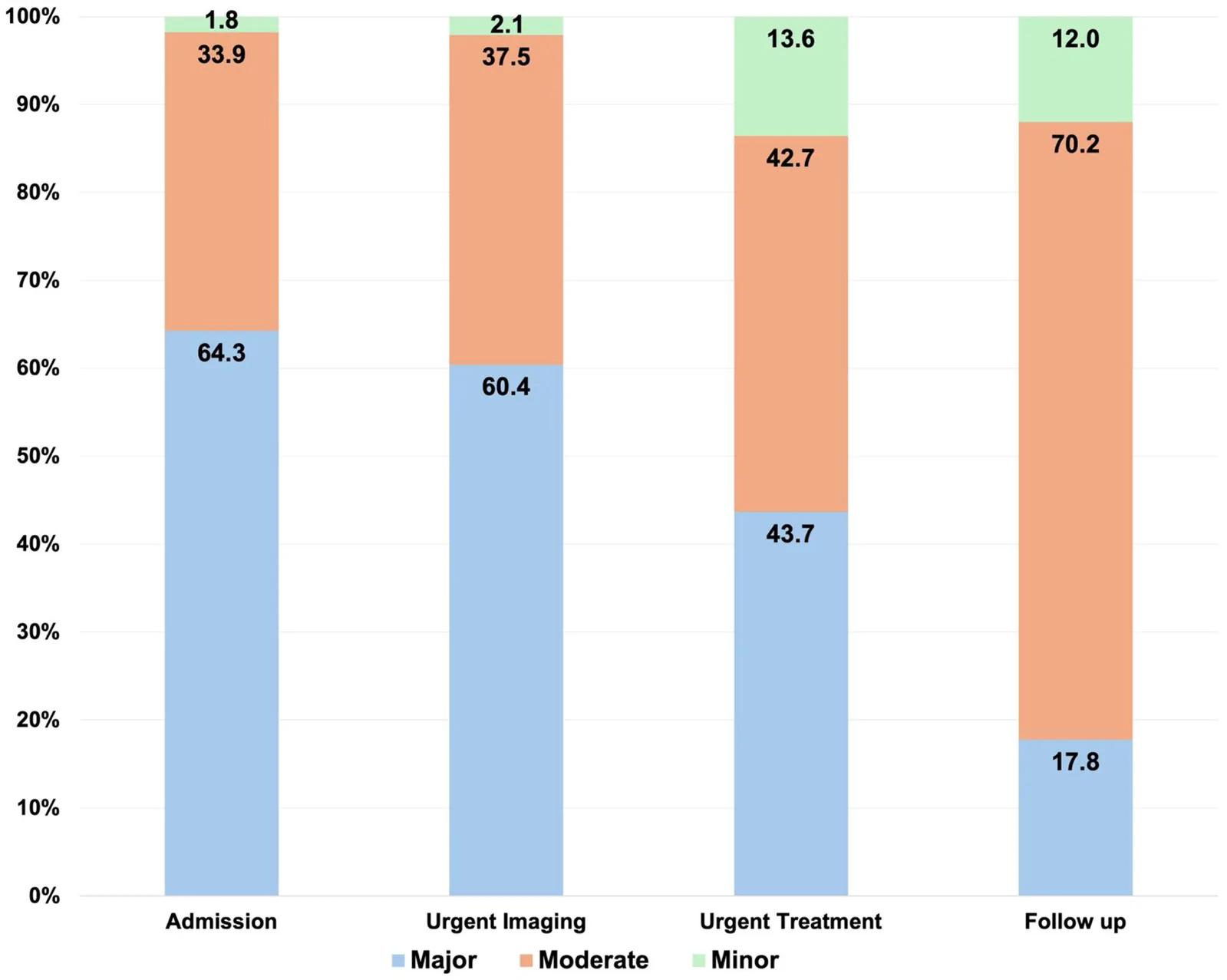
Distribution of Incidental Finding Severity Among Patients Receiving Each Management Action.

**Notes**: Percentages represent the distribution of incidental finding severity among patients who received each specific management action. Management actions were not mutually exclusive; patients could receive more than one management action. The y-axis represents the percentage of patients.

Incidental findings (IFs) were present in 1531 scans (54.9%). Of these, 6.1% were classified as major, 30.8% as moderate, and 63.2% as minor. Major and moderate findings together represent lesions considered clinically significant within our classification framework, while minor findings correspond primarily to benign or age-related abnormalities not typically requiring further intervention. Most patients with incidental findings had a single finding, with only a small proportion demonstrating multiple incidental findings (Supplementary Table 1). The distribution of IF categories is illustrated in Figure 2. Major IFs included suspected malignancy, aortic aneurysm, and acute appendicitis. Moderate IFs were mainly complex cysts, hernias, and bone lesions; minor IFs were mostly benign cysts and vascular calcifications. Hospital admission for IFs occurred in 3.7% of cases, specialist referral in 7.6%, and treatment initiation in 6.7%. Major IFs accounted for most admissions and urgent interventions (Figure 3). Management of the primary condition was delayed due to IFs in only 0.6% of cases (Table 2).

The most frequent IF categories were vascular calcifications (12%), bone lesions (10.6%) and hernias (9.1%) (Table 3).

**Table 3:** Incidental Findings Found in CT-KUB Scans

Category	Total N (%)	Subcategory	N (%)
Gastrointestinal	481 (20.1%)	Hernia	218 (45.3%)
		Diverticulosis	115 (23.9%)
		Colitis/BWT	52 (10.8%)
Genitourinary	373 (15.6%)	Contralateral Renal Stone	163 (43.7%)
		Renal Cyst	97 (26.0%)
		Adnexal Lesion	38 (10.2%)
Hepatobiliary	304 (12.7%)	Liver Lesion	187 (61.5%)
		Cholelithiasis	114 (37.5%)
		Cholecystitis	3 (1.0%)
Vascular	357 (14.9%)	Vascular Calcification	286 (80.1%)
		Organ Calcification	65 (18.2%)
		Pericardial Effusion	3 (0.8%)
Pulmonary	273 (11.4%)	Pulmonary Changes	215 (78.8%)
		Pleural Effusion	38 (13.9%)
		Pneumonia	6 (2.2%)
Musculoskeletal	259 (10.8%)	Bone Lesion	254 (98.1%)
		Spina Bifida	1 (0.4%)
Endocrine	81 (3.4%)	Adrenal Lesion	81 (100.0%)
Lymphatic	58 (2.4%)	Lymphadenopathy	58 (100.0%)
Infectious	10 (0.4%)	Pyelonephritis	5 (50.0%)
		Pyelonephritis/Cystitis	4 (40.0%)
		Cystitis	4 (40.0%)
Miscellaneous	198 (8.3%)	Other	178 (89.9%)
		Organomegaly	19 (9.6%)
		Nodules	1 (0.5%)

**Figure 4:**
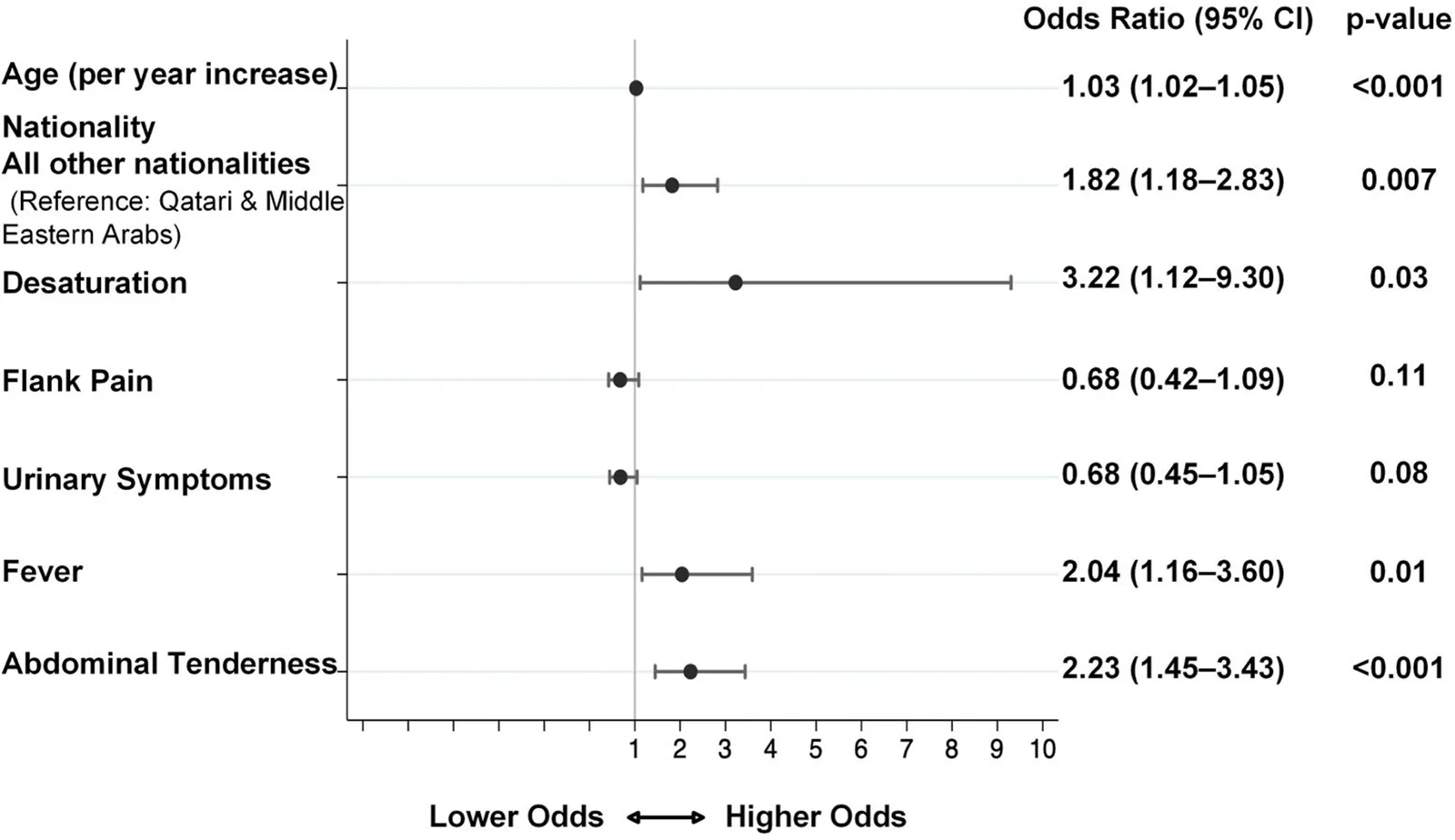
Factors Associated with Major Incidental Findings: Adjusted Odds Ratios from Multivariable Logistic Regression (n=2780).

**Notes**: The forest plot provides a graphical representation of adjusted odds ratios and 95% confidence intervals; the horizontal axis is presented for visual interpretation of lower versus higher odds. Model discrimination was assessed using the area under the receiver operating characteristic curve (AUC=0.720).

**Figure 5:**
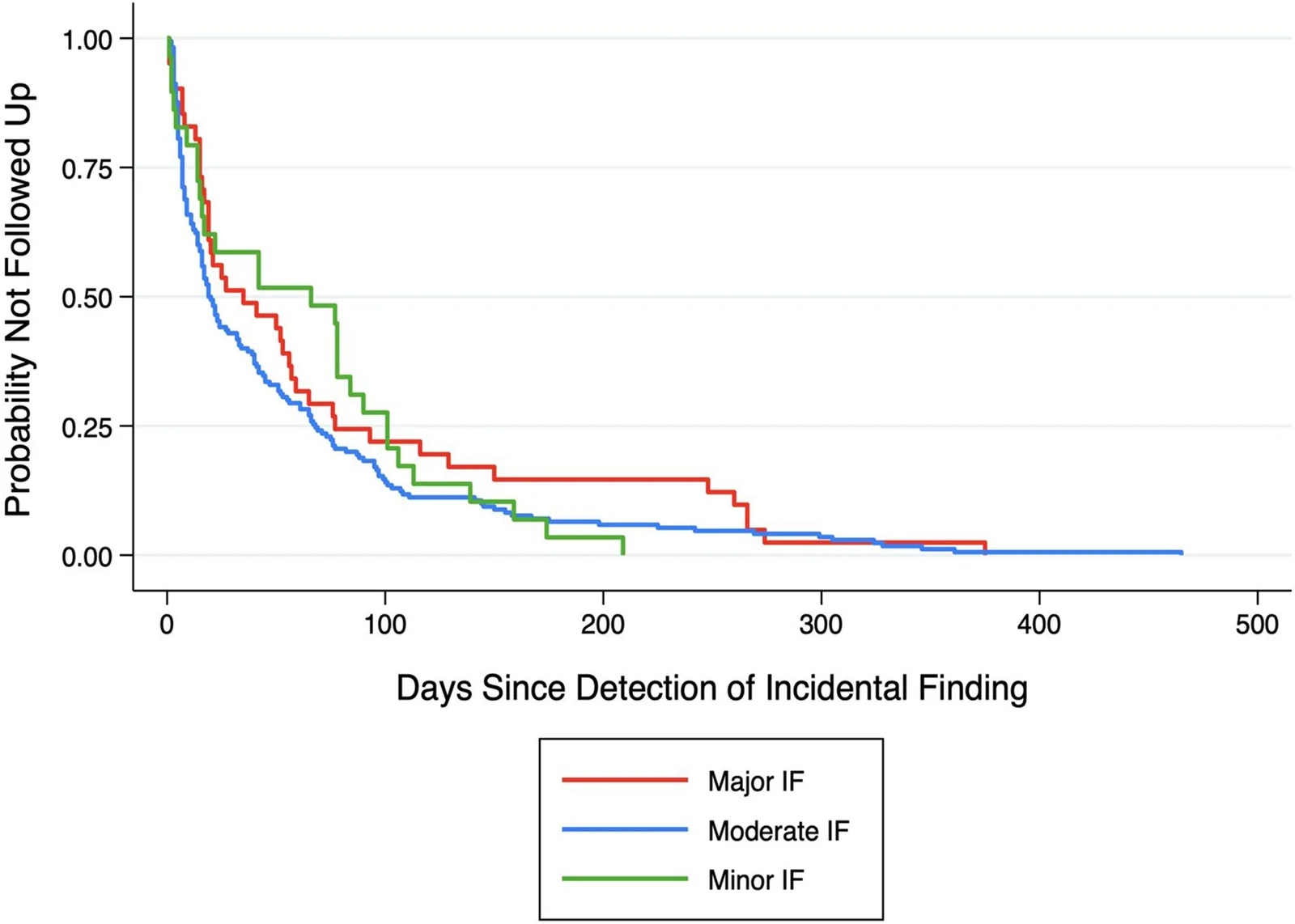
Kaplan–Meier Curves for Time to Follow-Up by Incidental Finding Severity.

**Notes**: The curves represent the probability of remaining without follow-up after detection of an incidental finding, stratified by highest-order incidental finding severity. Differences between groups were assessed using the Log rank test (p=0.29).

Multivariable logistic regression (Figure 4) showed that older age (aOR 1.03 per year), nationality classified as all other nationalities versus Qatari and Middle Eastern Arabs, fever, desaturation, and abdominal tenderness were independently associated with major IFs (all p<0.05). Bootstrap internal validation using 1000 resamples demonstrated limited model optimism (mean optimism=0.014). The apparent AUC was 0.720 and the optimism-corrected AUC was 0.706, with a mean calibration slope of 0.934, indicating limited overfitting and reasonable model stability. A sensitivity analysis excluding nationality yielded similar effect estimates for the remaining factors with only a modest reduction in model discrimination (Supplementary Table 2). No significant difference in time to follow-up was observed across IF severity categories (Figure 5).

## Discussion

In this large, diverse cohort from a tertiary emergency department in Qatar, we found that incidental findings (IFs) on CT KUB were common, affecting more than half of all scans. While most IFs were minor, a significant proportion were clinically relevant, requiring further intervention or specialist referral. Our findings highlight both the clinical and operational challenges posed by IFs in acute care settings.

The overall incidental findings rate in our study population was significantly higher than that reported in several prior CT KUB studies. A study from North America reported significant IFs in only 12.7% of 5383 scans, while another systematic review of 69 studies across 16 countries reported an IF rate of 31.3% in 1385 scans.^23^,^30^ The higher prevalence observed in our study could be multi-factorial and may not be limited to methodological differences such as the inclusion of minor and moderate findings rather than restricting the analysis to immediately actionable lesions. In addition, patient demographics being diverse and predominantly younger expatriate males undergoing high volumes of imaging, differences in disease burden and institutional reporting practices may further explain the discrepancies. Furthermore, co-occurrence of multiple incidental findings within the same patient was also uncommon in our cohort, with most patients demonstrating a single finding (Supplementary Table 1), which limits the feasibility of meaningful clustering analyses in this dataset.

It is important to distinguish between the prevalence of any incidental abnormality and the prevalence of clinically significant incidental findings. In the present study, the overall prevalence (54.9%) reflects all abnormalities reported in routine radiology reports, including benign or age-related findings such as vascular calcifications and degenerative skeletal changes. When applying our severity-based framework, clinically significant incidental findings correspond to the major and moderate categories, whereas minor findings largely represent benign conditions that typically do not require further diagnostic work-up. This distinction is consistent with prior epidemiological studies of incidental imaging findings, where reported prevalence varies substantially depending on whether all abnormalities or only clinically actionable lesions are included.^1^,^2^

These findings are consistent with broader emergency CT literature demonstrating considerable heterogeneity in the reported prevalence and classification of incidental findings across different clinical settings. Previous systematic reviews have emphasized that differences in study populations, imaging protocols, and definitions of clinically significant incidental findings contribute substantially to this variability, highlighting the need for standardized classification and reporting systems.^23^,^24^

The most frequent findings observed in our study included vascular calcifications, bone lesions, hernias, pulmonary changes, and liver findings, aligning with previous reports.^11^,^31^ CT KUB protocols are optimized for the detection of urolithiasis and are typically performed without intravenous contrast, which substantially limits soft-tissue characterization. As a result, incidental findings involving solid abdominal organs—particularly the liver, pancreas, and adrenal glands—may be under-detected or incompletely characterized on non-contrast imaging.^32^,^33^ Compared with contrast-enhanced abdominal CT, non-contrast CT has lower sensitivity for identifying complex renal cysts, pancreatic cystic lesions such as intraductal papillary mucinous neoplasms, and lipid-poor adrenal masses.^3^ This imaging limitation may partially explain the relatively low prevalence of certain complex lesions observed in our cohort. Therefore, the prevalence of clinically significant incidental findings reported in this study should be interpreted as conservative estimates.

Major incidental findings observed in our study such as abdominal aortic aneurysms (AAAs), acute appendicitis, and pancreatic masses, also align closely with the types of high-risk pathologies consistently highlighted in the existing literature.^3^ While the Log rank test did not demonstrate statistically significant differences in time-to-follow-up between incidental finding severity groups (p = 0.29), Kaplan–Meier curves were retained to illustrate overall follow-up dynamics across groups. Major IFs, though relatively uncommon, accounted for most admissions and urgent interventions. This finding emphasizes the importance of systematic approaches to triage and follow-up, as failure to address significant IFs can lead to adverse outcomes. Conversely, the high rate of minor IFs raises concerns about overdiagnosis, unnecessary investigations, and increased healthcare utilization. Our results suggest that structured reporting and clear communication pathways are essential to balance patient safety with resource stewardship.

Increasing age was significantly and independently associated with major IFs, consistent with prior findings. Samim et al reported that the prevalence of major IFs were approximately four times more common in individuals over 80 years of age as compared with younger patients.^28^ In our study, belonging to all other nationality groups, compared with Qatari and Middle Eastern Arab patients, as well as presenting with fever and oxygen desaturation, was also associated with higher odds of major incidental findings. These associations may reflect broader population-level risk patterns influenced by genetics, environmental, occupational or healthcare related factors. If validated externally, such associations could inform targeted imaging strategies, risk stratification and an implementation of early incidental finding risk flags to support clinical decision making in the emergency department.

The utilization of standardized framework for reporting incidental findings such as the guidelines by the ACR Incidental Findings Committee,^3^,^22^ can streamline clinical decision-making by linking lesion characteristics to evidence based management recommendations. In an emergency department setting, systematic application of such framework could facilitate more efficient triage, enabling emergency clinicians to distinguish patients who require immediate intervention from those appropriate for observation or safe discharge. The ACR guidelines further emphasize the significance of coordinated workflows, structured reporting phrasing, standardized reporting pathways between radiologists and emergency physicians, and reliable mechanisms for tracking follow up.^25^ The findings of our study support the relevance of such model, highlighting that responses tailored to the severity of incidental finding can optimize patient safety while avoiding unnecessary utilization of hospital resources.

From a health system perspective, these findings have significant operational implications. Radiology reporting templates may be strengthened by incorporating standardized grading of incidental finding severity along with corresponding management recommendations. Hospitals may further enhance patients care quality through the implementation of tracking systems to identify incidental findings to ensure that important findings are not missed post-discharge, a quality gap highlighted in prior practice audits.^8^ Moreover, refinement of CT KUB utilization guidelines, particularly for low-risk renal colic presentations, may help optimize diagnostic yield while promoting imaging stewardship. Evidence from regional studies, such as a report from Saudi Arabia demonstrated a 66.8% positive stone detection with CT KUB^34^ supports continued use of CT in appropriately selected cases while advocating the need for more defined, evidence based indications.

Our findings support the implementation of standardized, severity-based reporting for IFs, along with robust tracking systems to ensure appropriate follow-up. Emergency departments should consider adopting context-specific guidelines and decision-support tools to optimize imaging utilization and minimize unnecessary interventions. Future prospective studies should focus on developing and validating risk stratification approaches, evaluating long-term patient outcomes, and assessing the cost-effectiveness of different management strategies.

## Strengths and Limitations

This study is one of the largest series examining incidental findings on ED-performed CT KUB and is the first such analysis reported from Qatar. Key strengths include a large, and demographically diverse patient population, of the ability to link imaging findings to subsequent clinical management actions (including further imaging, treatment, and hospital admission), and the systematic classification of incidental findings according to clinical relevance.

Several limitations should also be acknowledged. Because this was a retrospective study including all eligible CT KUB examinations during the study period, no formal a priori sample size calculation was performed. The retrospective, single-center design limits causal inference and external validity. Reliance on radiology reports without a centralized image review may have introduced underreporting or inconsistency in the detection of subtle findings. However, in our institution CT examinations undergo a two-step reporting process, where a preliminary interpretation is generated by a board-certified radiologist during the emergency department encounter and subsequently finalized following a second review by another board-certified radiologist. While this workflow likely reduces variability related to individual reporting practices, the use of report-based data rather than direct image re-evaluation remains a limitation and may still allow some inter-observer variability in the detection of subtle incidental findings. In addition, missing or incomplete documentation may have biased estimates of subsequent management. The results may not be fully generalizable to institutions with different patient populations, imaging protocols, healthcare systems, or radiology reporting practices. Furthermore, we were unable to assess long-term outcomes for many incidentalomas.

## Conclusion

Our findings show that in a high-volume, diverse emergency department, over half of CT KUB scans revealed at least one incidental finding; however, most were minor, while a smaller proportion required urgent management or specialist referral. This high prevalence in a younger, multi-ethnic population challenges the assumption that incidental findings are mainly an issue for older or comorbid patients. These results highlight the need for tailored, context-specific protocols and underscore the importance of prospective multicenter studies to externally validate these findings and support standardized classification and management of incidental findings.

## Data Availability

The datasets generated and analyzed during the current study are not publicly available to protect the privacy and confidentiality of the research participants. However, the de-identified data are available from the corresponding author on reasonable request.

## References

[1] O’Sullivan JW, Muntinga T, Grigg S, Ioannidis JPA. Prevalence and outcomes of incidental imaging findings: umbrella review. *BMJ*. 2018;k2387. doi:10.1136/bmj.k2387 29914908 PMC6283350

[2] Lumbreras B, Donat L, Hernández-Aguado I. Incidental findings in imaging diagnostic tests: a systematic review. *BJR*. 2010;83(988):276–13. doi:10.1259/bjr/98067945 20335439 PMC3473456

[3] Berland LL, Silverman SG, Gore RM, et al. Managing incidental findings on abdominal CT: white paper of the ACR incidental findings committee. *J Am College Radiol*. 2010;7(10):754–773. doi:10.1016/j.jacr.2010.06.013 20889105

[4] Messersmith WA, Brown DFM, Barry MJ. The prevalence and implications of incidental findings on ED abdominal CT scans. *Am J Emerg Med*. 2001;19(6):479–481. doi:10.1053/ajem.2001.27137 11593466

[5] Yigit Y, Ayhan H. Incidental CT findings of patients who admitted to ER following a traffic accident. *Turk J Emerg Med*. 2014;14(1):9–14. doi:10.5505/1304.7361.2014.13284 27331159 PMC4909874

[6] Zagoria RJ. Retrospective view of “diagnosis of acute flank pain: value of unenhanced helical CT. *Am J Roentgenol*. 2006;187(3):603–604. doi:10.2214/AJR.06.0263 16928918

[7] Nash K, Hafeez A, Hou S. Hospital-acquired renal insufficiency. *Am J Kidney Dis*. 2002;39(5):930–936. doi:10.1053/ajkd.2002.32766 11979336

[8] Hanna TN, Shekhani H, Zygmont ME, Kerchberger JM, Johnson JO. Incidental findings in emergency imaging: frequency, recommendations, and compliance with consensus guidelines. *Emerg Radiol*. 2016;23(2):169–174. doi:10.1007/s10140-016-1378-1 26842832

[9] Liu W, Mortelé KJ, Silverman SG. Incidental extraurinary findings at MDCT urography in patients with hematuria: prevalence and impact on imaging costs. *Am J Roentgenol*. 2005;185(4):1051–1056. doi:10.2214/AJR.04.0218 16177432

[10] Fulgham PF, Assimos DG, Pearle MS, Preminger GM. Clinical effectiveness protocols for imaging in the management of ureteral calculous disease: AUA technology assessment. *J Urol*. 2013;189(4):1203–1213. doi:10.1016/j.juro.2012.10.031 23085059

[11] Thompson RJ, Wojcik SM, Grant WD, Ko PY. Incidental findings on CT scans in the emergency department. *Emerg Med Int*. 2011;2011:1–4. doi:10.1155/2011/624847 PMC3200145 22046542

[12] Bos D, Poels MMF, Adams HHH, et al. Prevalence, clinical management, and natural course of incidental findings on brain MR images: the population-based Rotterdam scan study. *Radiology*. 2016;281(2):507–515. doi:10.1148/radiol.2016160218 27337027

[13] Welch HG, Black WC. Overdiagnosis in Cancer. *JNCI J Nat Cancer Inst*. 2010;102(9):605–613. doi:10.1093/jnci/djq099 20413742

[14] Pandharipande PV, Reisner AT, Binder WD, et al. CT in the emergency department: a real-time study of changes in physician decision making. *Radiology*. 2016;278(3):812–821. doi:10.1148/radiol.2015150473 26402399

[15] Silverman SG, Israel GM, Herts BR, Richie JP. Management of the incidental renal mass. *Radiology*. 2008;249(1):16–31. doi:10.1148/radiol.2491070783 18796665

[16] Safari S, Dizaji SR, Yousefifard M, Taheri MS, Sharifi A. Prevalence and clinical significance of incidental findings in chest and abdominopelvic CT scans of trauma patients; A cross-sectional study. *Am J Emerg Med*. 2024;82:117–124. doi:10.1016/j.ajem.2024.06.008 38901332

[17] Bancos I, Prete A. Approach to the patient with adrenal incidentaloma. *J Clin Endocrinol Metab*. 2021;106(11):3331–3353. doi:10.1210/clinem/dgab512 34260734 PMC8530736

[18] Mayo-Smith WW, Song JH, Boland GL, et al. Management of incidental adrenal masses: a white paper of the ACR incidental findings committee. *J Am College Radiol*. 2017;14(8):1038–1044. doi:10.1016/j.jacr.2017.05.001 28651988

[19] Clark TJ, Coats G. Adherence to ACR incidental finding guidelines. *J Am College Radiol*. 2016;13(12):1530–1533. doi:10.1016/j.jacr.2016.05.008 27319370

[20] Lai W-A, Liu P-H, Tsai M-J, Huang YC. Frequency, recognition, and potential risk factors of incidental findings on trauma computed tomography scans: a cross-sectional study at an urban level one trauma center. *J Acute Med*. 2020;10(3):106–114. doi:10.6705/j.jacme.20200910(3).0002 33209569 PMC7662100

[21] Davenport MS. Incidental findings and low-value care. *Am J Roentgenol*. 2023;221(1):117–123. doi:10.2214/AJR.22.28926 36629303

[22] Herts BR, Silverman SG, Hindman NM, et al. Management of the incidental renal mass on CT: a white paper of the ACR incidental findings committee. *J Am College Radiol*. 2018;15(2):264–273. doi:10.1016/j.jacr.2017.04.028 28651987

[23] Evans CS, Arthur R, Kane M, et al. Incidental radiology findings on computed tomography studies in emergency department patients: a systematic review and meta-analysis. *Ann Emerg Med*. 2022;80(3):243–256. doi:10.1016/j.annemergmed.2022.03.027 35717273

[24] Nagasawa K, Nihashi T, Iwata M, Terasawa T. Prevalence of incidental findings requiring interventions in CT for trauma patients in the emergency department: a systematic review and meta-analysis. *BMJ Open*. 2026;16:e103045. doi:10.1136/bmjopen-2025-103045 PMC13110626 41991266

[25] Khosa F, Krinsky G, Macari M, Yucel EK, Berland LL. Managing incidental findings on abdominal and pelvic CT and MRI, Part 2: white paper of the ACR incidental findings committee II on vascular findings. *J Am College Radiol*. 2013;10(10):789–794. doi:10.1016/j.jacr.2013.05.021 24091049

[26] Kirkpatrick IDC, Brahm GL, Mnatzakanian GN, Hurrell C, Herts BR, Bird JR. Recommendations for the management of the incidental renal mass in adults: endorsement and adaptation of the 2017 ACR incidental findings committee white paper by the canadian association of radiologists incidental findings working group. *Can Assoc Radiol J*. 2019;70(2):125–133. doi:10.1016/j.carj.2019.03.002 30962049

[27] MacMahon H, Naidich DP, Goo JM, et al. Guidelines for management of incidental pulmonary nodules detected on CT images: from the Fleischner Society 2017. *Radiology*. 2017;284(1):228–243. doi:10.1148/radiol.2017161659 28240562

[28] Goodman A. The development of the Qatar healthcare system: a review of the literature. *IJCM*. 2015;06(03):177–185. doi:10.4236/ijcm.2015.63023

[29] Kodumayil SA, Kodumayil A, Thomas SA, et al. Q-DEPICT: Qatar determining emergency physician incidence of COVID-positive testing. *Qatar Med J*. 2021;2021. doi:10.5339/qmj.2021.44 PMC8501270 34660215

[30] Samim M, Goss S, Luty S, Weinreb J, Moore C. Incidental findings on CT for suspected renal colic in emergency department patients: prevalence and types in 5383 consecutive examinations. *J Am College Radiol*. 2015;12(1):63–69. doi:10.1016/j.jacr.2014.07.026 25557571

[31] Belloni E, Tentoni S, Fiorina I, et al. Reported and unreported potentially important incidental findings in urgent nonenhanced abdominal CT for renal colic. *Med Princ Pract*. 2021;30(4):355–360. doi:10.1159/000515852 33721865 PMC8436713

[32] Boland GW, Blake MA, Hahn PF, Mayo-Smith WW. Incidental adrenal lesions: principles, techniques, and algorithms for imaging characterization. *Radiology*. 2008;249(3):756–775. doi:10.1148/radiol.2493070976 19011181

[33] Megibow AJ, Baker ME, Morgan DE, et al. Management of incidental pancreatic cysts: a white paper of the ACR incidental findings committee. *J Am Coll Radiol*. 2017;14(7):911–923. doi:10.1016/j.jacr.2017.03.010 28533111

[34] Aljawad M, Alaithan FA, Bukhamsin BS, Alawami AA. Assessing the diagnostic performance of CT in suspected urinary stones: a retrospective analysis. *Cureus*. 2023;15(4):1. 10.7759/cureus.37699 PMC10191237 37206506

